# CCP-YOLO: An Improved YOLOv11n Algorithm for Steel Surface Defect Detection

**DOI:** 10.3390/s26154920

**Published:** 2026-08-04

**Authors:** Li Xiao, Pengyang Li, Caidong Wang, Huadong Zheng, Yapeng Xu

**Affiliations:** 1 School of Computer Science and Artificial Intelligence, Zhengzhou University of Light Industry, Zhengzhou 450002, China; xiaoli2010@zzuli.edu.cn; 2 College of Mechanical and Electrical Engineering, Zhengzhou University of Light Industry, Zhengzhou 450002, China; lpyssj@163.com (P.L.); hurd_cheng@163.com (H.Z.); yapengxu@zzuli.edu.cn (Y.X.)

**Keywords:** steel surface defect detection, multi-scale feature extraction, adaptive feature fusion, context aggregation, loss function

## Abstract

**Highlights:**

**What are the main findings?**

- CCP-YOLO improves YOLOv11n for steel surface defect detection by enhancing multi-scale feature extraction, heterogeneous feature fusion, spatial-detail-preserving context aggregation, and bounding box regression.
- The proposed model achieves 80.2% mAP50 on the NEU-DET dataset, improving the YOLOv11n baseline by 4.1 percentage points, while maintaining 2.7 M parameters, 6.6 GFLOPs, and an inference speed of 133.14 FPS.

**What is the implication of the main findings?**

- CCP-YOLO provides an accurate and lightweight solution for detecting small, multi-scale, and irregular steel surface defects in industrial inspection scenarios, and its improved performance on both NEU-DET and GC10-DET demonstrates strong generalization potential for practical steel surface quality inspection systems.

**Abstract:**

Steel surface defect detection remains challenging due to difficulties in multi-scale feature extraction, limited effectiveness of heterogeneous feature fusion, and loss of spatial detail information. To address these issues, this paper proposes CCP-YOLO, an improved steel surface defect detection algorithm based on YOLOv11n. The proposed method introduces four targeted improvements: (1) a Multi-Scale Dilated Reparameterization module (C3k2_MSD) for enhanced multi-scale feature extraction via a three-branch parallel reparameterization architecture; (2) an Interactive Adaptive Feature Fusion Module (IAFM) for effective integration of heterogeneous features; (3) a Progressive Shared-Weight Context Aggregation (PSWCA) module replacing the original SPPF structure to preserve spatial detail; and (4) a Wise-Inner-MPDIoU fusion loss function for improved bounding box regression accuracy and stability. Experimental results on the NEU-DET dataset demonstrate that CCP-YOLO achieves an mAP50 of 80.2%, representing a 4.1 percentage-point improvement over the YOLOv11n baseline, with a recall of 0.754, 2.7 M parameters, 6.6 GFLOPs, and an inference speed of 133.14 FPS. Further validation on the GC10-DET dataset confirms a 3.7 percentage-point improvement in mAP50. These results indicate that CCP-YOLO effectively enhances detection accuracy while maintaining computational efficiency, demonstrating strong potential for real-world industrial deployment.

## 1. Introduction

Steel is an indispensable engineering material widely used in automotive manufacturing, shipbuilding, mechanical engineering, and aerospace industries. However, surface defects inevitably occur during steel production owing to variations in raw material quality, equipment conditions, and manufacturing processes. These defects can significantly deteriorate surface quality, corrosion resistance, and fatigue strength. Conventional manual inspection methods suffer from inherent limitations, such as high subjectivity, low efficiency, and poor reliability, making them inadequate for the high-precision, high-throughput inspection requirements of modern continuous production lines. Consequently, deep learning-based steel surface defect detection has attracted increasing research attention and has become an important development direction in intelligent manufacturing [1,2].

Current deep learning-based object detection algorithms are broadly categorized into two paradigms: single-stage detectors and two-stage detectors. Representative single-stage algorithms, such as SSD [3] and YOLO [4], directly generate detection results and offer the advantage of fast inference speed, albeit at the cost of relatively limited detection accuracy. In contrast, two-stage methods, exemplified by Faster R-CNN [5], achieve superior detection accuracy through region proposal generation followed by refined classification and regression. However, this improvement comes at the expense of substantially higher computational complexity. Industrial steel surface inspection on high-throughput continuous production lines places simultaneous demands on detection accuracy and real-time processing capability. High detection accuracy is essential for reducing missed detections and false alarms, whereas sufficient inference efficiency is required to support continuous online inspection without interrupting the production process. Nevertheless, existing detection methods still face considerable challenges when dealing with complex steel surface defects, such as inadequate recognition of small targets, insufficient multi-scale feature representation, and relatively high false-positive and false-negative rates. These limitations restrict their practical application in industrial inspection systems. Therefore, improving detection accuracy and robustness while maintaining computational efficiency and real-time performance remains an important research objective. To this end, YOLOv11n, as a next-generation lightweight single-stage detector, has achieved substantial improvements in detection accuracy over earlier single-stage methods while maintaining low computational overhead and strong feature representation capability, providing a solid architectural foundation for industrial defect detection. Nevertheless, its performance in multi-scale feature extraction and feature fusion still has room for further improvement.

In response to these challenges, numerous researchers have proposed various improvements to existing object detection frameworks. For instance, Chu et al. [6] proposed YOLO-SDS, a lightweight strip steel surface defect detection network based on an improved YOLOv8 framework, which improves multi-scale feature extraction and occluded-defect representation through lightweight optimization of the backbone and neck networks. Chen et al. [7] proposed DCAM-Net for rapid strip steel surface defect detection, enhancing defect feature extraction and localization performance under low-contrast and irregular defect conditions through image enhancement, deformable convolution, and attention mechanisms. Zhao et al. [8] designed a Dual Feature Pyramid Network (DFPN) with a decoupled head structure that separates regression and classification tasks; however, its performance remains limited when detecting defects with blurred boundaries or extremely small sizes. Zhao et al. [9] proposed the MSAF-YOLOv8n algorithm, which improves receptive field adaptation and multi-scale feature fusion strategies to enhance model adaptability while maintaining computational efficiency. Ma et al. [10] proposed a lightweight steel surface defect detection algorithm based on an improved YOLOv8 framework, which reduces model parameters and computational complexity through lightweight backbone optimization, attention-guided feature enhancement, and improved bounding box regression. Nevertheless, its lightweight design may still limit the representation of complex multi-scale defect features. He et al. [11] constructed a multi-level feature fusion network that strengthens the integration of features across different hierarchical levels; however, the method relies on fixed-weight fusion strategies and lacks an adaptive mechanism for modulating the importance of heterogeneous semantic layers. Liao et al. [12] proposed YOLOv10n-SFDC, a lightweight steel surface defect detection network that improves feature extraction, feature fusion, and bounding box regression, thereby enhancing detection accuracy for complex surface defects. Although these studies have achieved encouraging results, several limitations remain. First, most existing feature extraction modules predominantly employ fixed receptive-field designs, which may restrict their ability to represent steel surface defects with substantial scale variation. Second, conventional feature fusion methods based on direct concatenation or fixed-weight summation do not adaptively account for the relative importance of features from different semantic levels. Although attention mechanisms have been introduced into some feature-fusion necks, they commonly perform channel-wise or spatial-wise recalibration on already aggregated features. Such operations may enhance feature selection, but they do not necessarily model the semantic discrepancy and complementary interaction between low-level spatial details and high-level semantic representations. This limitation may reduce fusion effectiveness when defect appearance varies considerably in scale, morphology, and background texture. Third, the fixed pooling operations employed in Spatial Pyramid Pooling–Fast (SPPF) may cause spatial-detail loss during contextual information aggregation, thereby affecting the localization of small defects. Therefore, further improvements in multi-scale feature extraction, cross-level semantic interaction, and spatial-detail-preserving context aggregation are needed for steel surface defect detection under complex industrial conditions.

The proposed C3k2_MSD strategy differs from structural reparameterization modules previously adopted within the YOLO family, such as RepConv in YOLOv7 [13] and RepNCSPELAN in YOLOv9 [14], as well as generic designs such as RepVGG [15] and the Diverse Branch Block (DBB) [16]. Many representative structural reparameterization modules collapse a group of parallel training-time branches associated with a common target receptive-field scale into an equivalent convolution, primarily to reduce inference-time overhead. In contrast, C3k2_MSD combines a three-branch multi-scale architecture with branch-internal structural reparameterization. Specifically, the outer MSD unit comprises a standard 3 × 3 convolution branch, a DRB_5_ branch, and a DRB_7_ branch. During deployment, only the internal training-time branches of DRB_5_ and DRB_7_ are independently collapsed into equivalent 5 × 5 and 7 × 7 depthwise convolutions, respectively, while the standard 3 × 3 branch remains unchanged. The three outer scale-specific branches continue to operate in parallel and are subsequently fused through channel concatenation and a 1 × 1 convolution. Therefore, the main distinction lies in embedding structurally reparameterizable operators within two branches of parallel multi-scale architecture, rather than applying reparameterization to all three scale-specific branches. This design preserves receptive-field diversity across scales while reducing the inference overhead associated with the internal multi-branch structures of DRB_5_ and DRB_7_.

To address the aforementioned issues, this paper proposes an improved steel surface defect detection algorithm based on YOLOv11n, termed CCP-YOLO. Through the collaborative design of multiple targeted improvements, the proposed algorithm effectively enhances detection accuracy and robustness. The main contributions of this work are as follows:

(1) A Multi-Scale Dilated Reparameterization feature extraction module (C3k2_MSD) is proposed. The module adopts a multi-path parallel architecture combined with structural reparameterization to alleviate the limitation of fixed receptive fields in conventional feature extraction modules and to enhance the network’s capability of capturing defect features across multiple spatial scales.

(2) An Interactive Adaptive Feature Fusion Module (IAFM) is designed to improve the integration of heterogeneous hierarchical features. The module constructs a three-stage fusion architecture comprising semantic aggregation, branch-aware adaptive weighting, and semantic reshaping. By combining channel-wise recalibration with cross-branch residual interaction, IAFM facilitates complementary information exchange between low-level spatial details and high-level semantic representations, thereby improving feature discriminability under complex background conditions.

(3) A Progressive Shared-Weight Context Aggregation (PSWCA) module is designed to improve multi-scale contextual modeling. The module constructs a progressive context aggregation architecture in which shared convolutional kernels with varying dilation rates replace the fixed pooling operations of conventional SPPF, thereby enabling parameter-efficient and effective multi-scale context aggregation.

(4) A Wise-Inner-MPDIoU fusion loss function is constructed. To address the issues of inconsistent annotation quality, insufficient adaptability of IoU computation, and limited geometric modeling of complex defect shapes in steel surface defect detection, the proposed loss function integrates a quality-aware mechanism, an auxiliary bounding box mechanism, and corner-point distance constraints to achieve multi-level collaborative optimization, thereby improving the accuracy and stability of bounding box regression.

## 2. YOLOv11 Object Detection Algorithm

**Figure 1:**
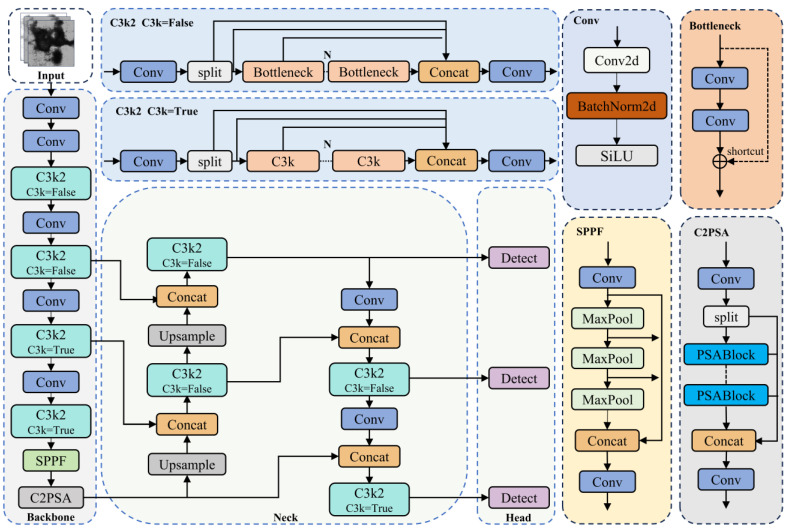
Network architecture of YOLOv11n.

YOLOv11 is a lightweight object detection framework that incorporates architectural refinements and parameter optimizations over YOLOv8. As shown in Figure 1, the model adopts a four-stage architecture consisting of the input module, backbone network, neck network, and detection head. In the backbone network, the C3k2 module replaces the original C2f module to enhance feature extraction capability through flexible parameter configurations. In addition, a C2PSA module is integrated after the SPPF layer to enhance the model’s attention to salient regions and improve feature representation quality. The neck network retains the FPN-PAN fusion architecture, employing a bidirectional feature propagation mechanism to effectively integrate multi-scale semantic features and spatial information. The detection head adopts depthwise separable convolutions and an anchor-free detection mechanism, reducing the number of parameters and the computational cost while maintaining detection accuracy.

## 3. CCP-YOLO Algorithm

**Figure 2:**
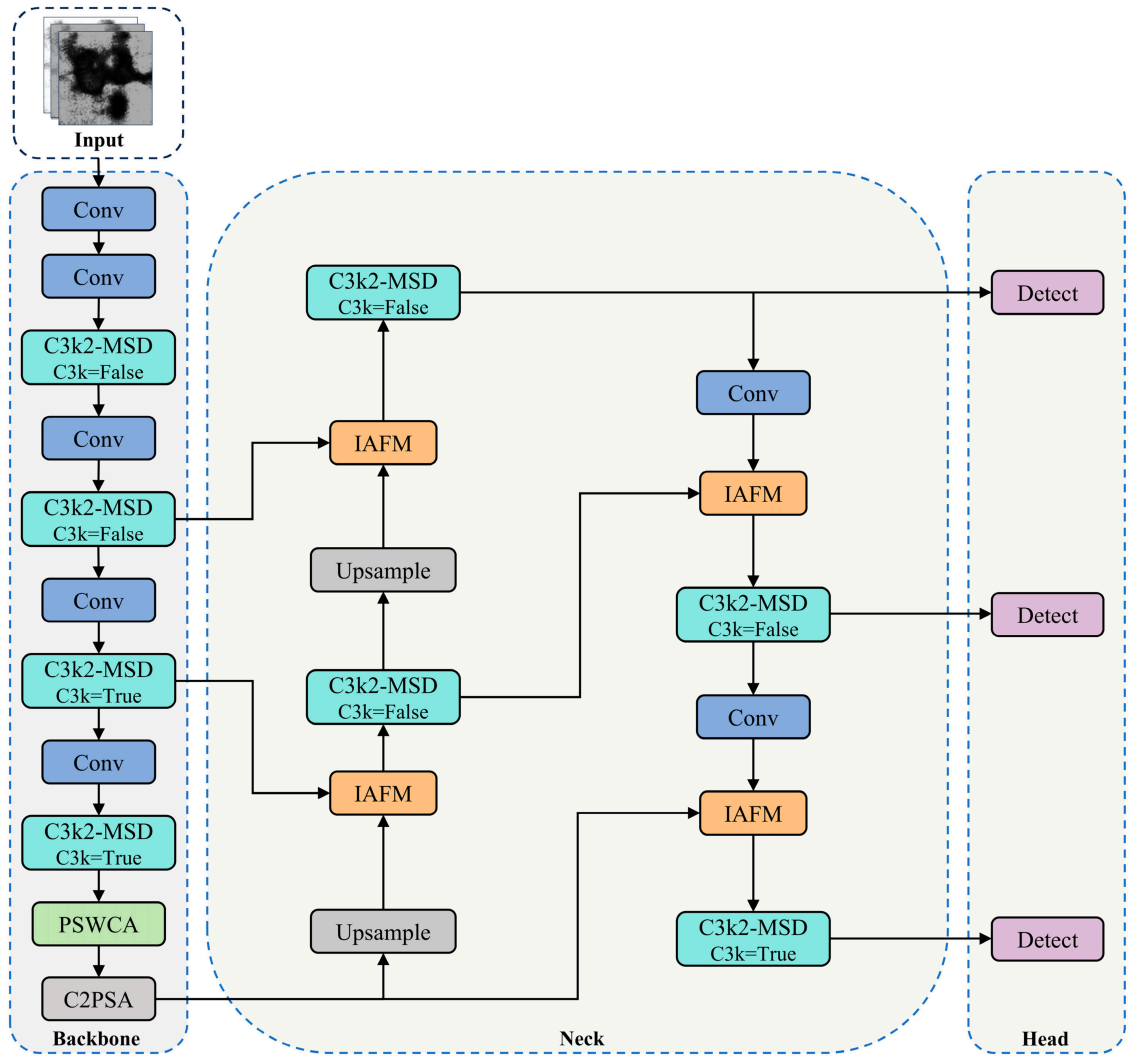
Network architecture of CCP-YOLO.

Building upon the YOLOv11n architecture, this paper proposes the CCP-YOLO detection algorithm, as shown in Figure 2. The proposed method incorporates four main improvements to enhance feature representation, feature fusion, contextual modeling, and localization accuracy. Specifically, the C3k2_MSD module is introduced into both the backbone and neck networks to strengthen multi-scale feature extraction capability. The IAFM is incorporated at the feature fusion stage to achieve adaptive fusion of heterogeneous features. The original SPPF module is replaced by the PSWCA module to improve global contextual feature aggregation through a progressive shared-weight mechanism. In addition, a Wise-Inner-MPDIoU fusion loss function is designed to optimize bounding box regression accuracy.

### 3.1. Multi-Scale Dilated Reparameterization Feature Extraction Module

Steel surface defects exhibit pronounced multi-scale distribution characteristics and complex morphological variations. In industrial inspection scenarios, conventional convolutional neural networks face the inherent limitation that fixed convolutional receptive fields cannot simultaneously capture defect patterns across multiple spatial scales [17]. Small receptive fields fail to capture the complete contours of large-scale defects, whereas large receptive fields tend to suppress or lose critical fine-grained details of small-scale defects. Furthermore, single-scale feature extraction strategies may be insufficient to distinguish subtle defects from background texture patterns on steel surfaces. Here, subtle defects refer to low-saliency defect regions characterized by one or more of the following properties: weak intensity or texture contrast relative to the surrounding surface, indistinct boundaries, or limited spatial extent. To address this limitation, this paper proposes the C3k2_MSD Multi-Scale Dilated Reparameterization feature extraction module. The module adopts a Multi-Scale Dilated (MSD) architecture as the core mechanism and incorporates the Dilated Reparameterization Block (DRB) [18] technique to enhance both feature representation capability and computational efficiency. Through parallel multi-path feature extraction and adaptive receptive field modeling, the proposed module improves the network’s ability to recognize defects with diverse scales and morphological characteristics.

**Figure 3:**
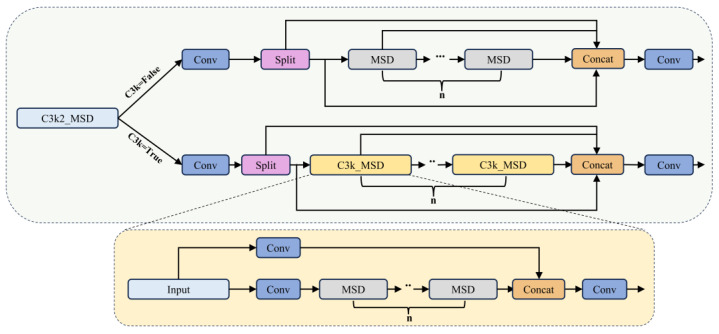
Configuration architecture of the C3k2_MSD module.

The C3k2_MSD module inherits the Cross Stage Partial (CSP) network design philosophy [19] and achieves efficient feature extraction through branch-wise processing and feature fusion. As shown in Figure 3, the module supports two configuration modes. When C3k = False, a compact architecture directly employing MSD units is adopted, making it suitable for computationally constrained scenarios. When C3k = True, a deeper C3k_MSD structure is employed, which enhances feature representation capability through increased network depth and is therefore more appropriate for complex defect recognition tasks. The module adopts a CSP-style branch architecture. For the C3k = False configuration, the input feature is first projected through a 1 × 1 convolution and then divided into two feature subsets. One subset bypasses the repeated MSD units and is routed to the concatenation stage, whereas the other is sequentially processed by n MSD units. For the C3k = True configuration, the processing path consists of repeated C3k_MSD blocks. As illustrated by the enlarged C3k_MSD structure in Figure 3, both internal subbranches include a convolutional projection. One subbranch bypasses the subsequent MSD stack and proceeds to the concatenation stage, while the other undergoes cascaded MSD processing. Therefore, the bypass branch refers to a feature path that bypasses the MSD transformations after convolutional projection, rather than an identity mapping of the original input. Finally, the outputs of the branches are concatenated and fused through a 1 × 1 convolution to produce the target output channels.

**Figure 4:**
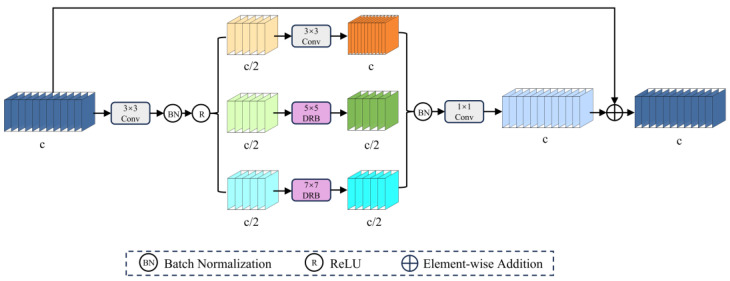
MSD multi-scale feature extraction architecture.

Within the C3k2_MSD module, the core structure employs the MSD multi-scale mechanism, in which two branches incorporate DRB reparameterization technology. The MSD structure is shown in Figure 4.

The MSD mechanism adopts a three-branch parallel architecture to enable discriminative feature extraction across defects of different scales. The input feature x is first subjected to channel dimensionality reduction via a 3 × 3 convolution and then routed into three parallel processing paths. The first path employs a standard 3 × 3 convolution to extract local fine-grained features with a relatively small receptive field, specifically targeting small-scale defects such as pitting. The second path integrates a DRB module with a kernel size of 5, capturing features of medium-scale defects such as scratches through moderate receptive field expansion. The third path integrates a DRB module with a kernel size of 7, providing the largest receptive field coverage for detecting large-area defects such as patches and cracks. The outputs of the three paths are fused via channel concatenation, integrated through a 1 × 1 convolution, and combined with the original input via a residual connection to achieve adaptive multi-scale feature fusion. The mathematical formulation of the MSD multi-scale architecture is expressed as follows:

(1) $$C_{1}(x)=\mathrm{ReLU}(\mathrm{BN}(Conv_{3\times 3}(x)))$$

(2) $$C_{21}(x)=Conv_{3\times 3}^{\left(d=1\right)}(C_{1}(x))$$

(3) $$C_{22}(x)=DRB_{5}(C_{1}(x))$$

(4) $$C_{23}(x)=DRB_{7}(C_{1}(x))$$

(5) $$MSD(x)=Conv_{1\times 1}(\mathrm{BN}(Concat(\left[C_{21}(x),C_{22}(x),C_{23}(x)\right])))+x$$

where $Conv_{3\times 3}(\cdot )$ denotes the standard 3 × 3 convolution, $Conv_{3\times 3}^{\left(d\right)}(\cdot )$ denotes the 3 × 3 convolution with dilation rate *d*, $DRB_{k}(\cdot )$ denotes the Dilated Reparameterization Block with kernel size *k*, and Concat(⋅) denotes the channel concatenation operation.

**Figure 5:**
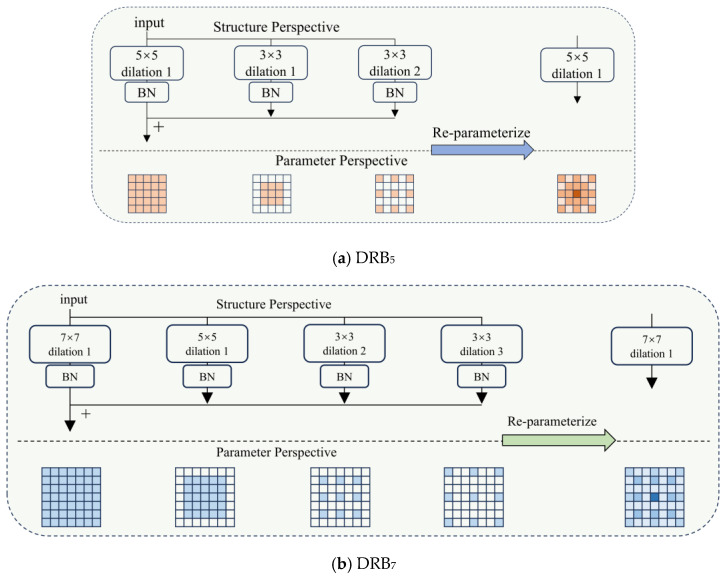
Analytical reparameterization of the DRB modules from training-time multi-branch structures to deployment-time single depthwise convolutions: (**a**) DRB_5_ converted into an equivalent 5 × 5 depthwise convolution; (**b**) DRB_7_ converted into an equivalent 7 × 7 depthwise convolution.

Within the MSD architecture, structural reparameterization based on the DRB design [20] is applied independently to the DRB_5_ and DRB_7_ branches. During training, DRB_5_ comprises three parallel depthwise convolution–batch normalization branches with kernel-size and dilation-rate pairs of (5, 1), (3, 1) and (3, 2), whereas DRB_7_ comprises four branches configured as (7, 1), (5, 1), (3, 2), and (3, 3). During deployment, the batch normalization parameters of each branch are absorbed into the corresponding convolution. The resulting kernels are expanded according to their dilation rates, zero-padded and center-aligned to a common spatial support, and then summed together with their biases. Consequently, DRB_5_ and DRB_7_ are converted into equivalent 5 × 5 and 7 × 7 depthwise convolutions, respectively. Figure 5 illustrates the training-time multi-branch structures and deployment-time equivalents of both modules. Under fixed inference-mode batch normalization statistics, the converted convolutions are mathematically equivalent to the sums of their corresponding linear branches, apart from possible floating-point rounding errors.

The following analytical reparameterization procedure applies to both DRB_5_ and DRB_7_. Let a training-time DRB contain M parallel depthwise convolution–batch normalization branches. The output of the *i*-th branch can be expressed as

(6) $$Y_{i}={\mathrm{BN}}_{i}(W_{i}{*}_{r_{i}}X+b_{i}),$$

where *W_i_*, *b_i_*, *k_i_*, and *r_i_* denote the convolutional kernel, convolutional bias, kernel size, and dilation rate of the *i*-th branch, respectively, and ${*}_{r_{i}}$ denotes depthwise convolution with dilation rate *r_i_*. During inference, the batch normalization parameters are absorbed into the corresponding convolutional kernel and bias. For the *c*-th channel, the fused parameters are given by

(7) $${\hat{W}}_{i,c}=\frac{\gamma_{i,c}}{\sqrt{\sigma_{i,c}^{2}+\varepsilon }}W_{i,c},$$

(8) $${\hat{b}}_{i,c}=\beta_{i,c}+\frac{\gamma_{i,c}}{\sqrt{\sigma_{i,c}^{2}+\varepsilon }}\left(b_{i,c}-\mu_{i,c}\right),$$

where $\mu_{i,c}$, $\sigma_{i,c}^{2}$, $\gamma_{i,c}$, and $\beta_{i,c}$ are the running mean, running variance, scaling parameter, and shifting parameter of the corresponding batch normalization layer, respectively, and ε is the numerical stabilization constant. In the training-time implementation of the DRB, the convolutional bias is disabled; therefore, $b_{i,c}=0$.

For a branch with kernel size $k_{i}$ and dilation rate $r_{i}$, the effective kernel size is

(9) $$s_{i}=(k_{i}-1)\;r_{i}+1.$$

To enable parameter summation across branches with different kernel sizes and dilation rates, the fused kernel ${\hat{W}}_{i}$ is first expanded into an equivalent dense kernel by inserting $r_{i}-1$ zeros between adjacent kernel elements. The expanded kernel is then symmetrically zero-padded and center-aligned to the target kernel size K×K. Denoting this transformation by $\Gamma_{K}^{\left(k_{i},r_{i}\right)}(\cdot )$, the equivalent deployment-time kernel and bias are obtained as

(10) $$W_{\mathrm{eq}}=\sum_{i=1}^{M}\Gamma_{K}^{\left(k_{i},r_{i}\right)}\left({\hat{W}}_{i}\right)$$

(11) $$b_{\mathrm{eq}}=\sum_{i=1}^{M}{\hat{b}}_{i}$$

Accordingly, the training-time multi-branch computation can be represented during deployment by a single equivalent depthwise convolution:

(12) $$\sum_{i=1}^{M}{\mathrm{BN}}_{i}\left(W_{i}{*}_{r_{i}}X+b_{i}\right)=W_{\mathrm{eq}}*X+b_{\mathrm{eq}}$$

For DRB_5_, the transformed branch kernels are aligned to a common 5 × 5 support, whereas those of DRB_7_ are aligned to a common 7 × 7 support. The conversion is performed independently within the two DRB branches. The standard 3 × 3 branch remains unchanged, and the three outer scale-specific branches of the MSD unit continue to operate in parallel during inference.

### 3.2. Interactive Adaptive Feature Fusion Module

During multi-level feature fusion, the defect-related information encoded in different semantic layers exhibits intrinsic heterogeneity, manifested as disparities in semantic granularity and inter-layer semantic interference. This heterogeneity hinders the effective utilization of critical information, as informative features tend to be suppressed while redundant responses are amplified. Under complex steel surface conditions, texture-rich backgrounds and interfering patterns further intensify information redundancy, making it easy for genuine defect features to be overwhelmed by background noise. Conventional feature fusion methods, such as simple concatenation or fixed-weight summation, lack the capability to adaptively quantify the relative importance of different semantic layers, thereby limiting the network’s adaptability to the detection of various defect types [21].

**Figure 6:**
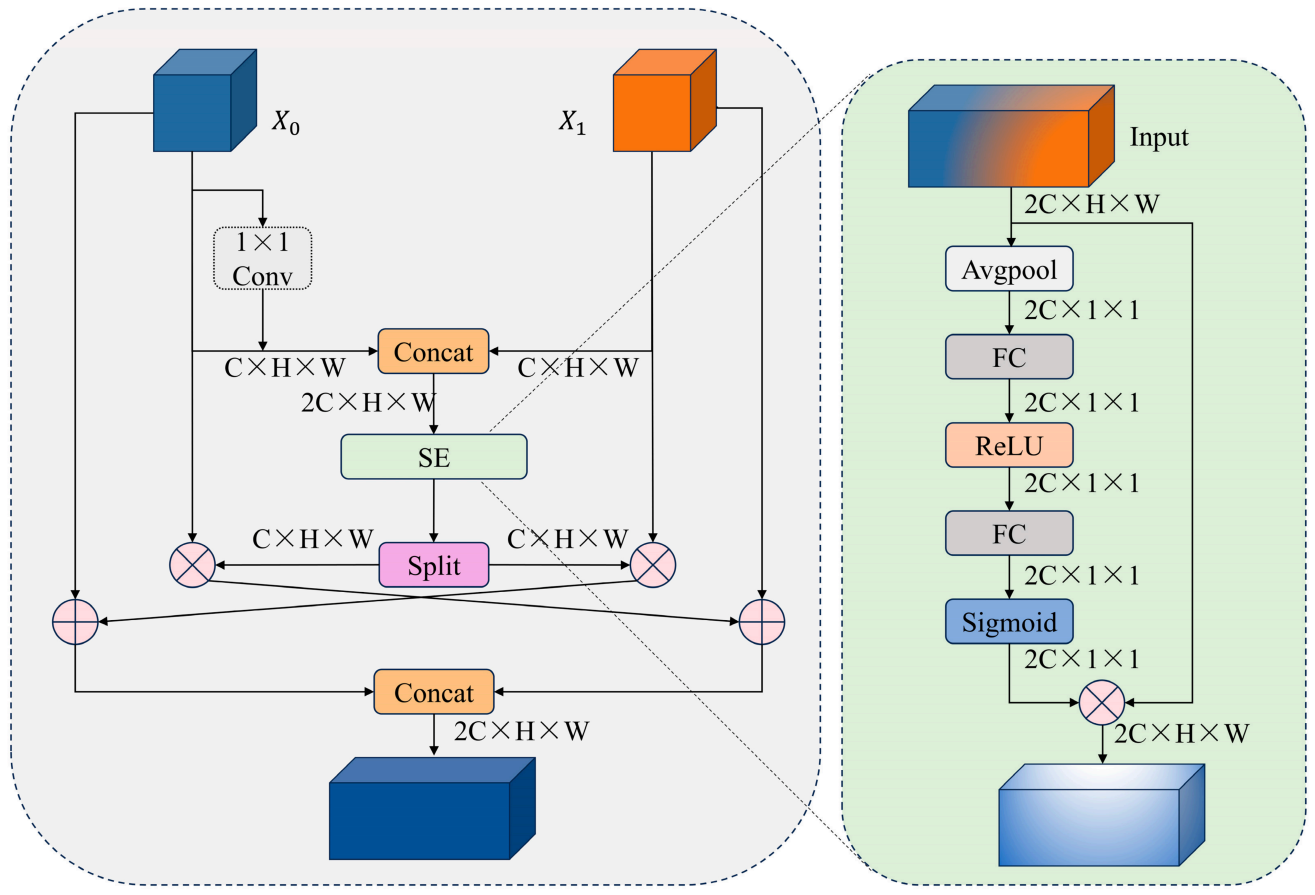
Architecture of the Interactive Adaptive Feature Fusion (IAFM) module.

To address these issues, this paper proposes the Interactive Adaptive Feature Fusion Module (IAFM). The module constructs a three-stage fusion architecture comprising semantic aggregation, adaptive weighting, and semantic reshaping. In addition, it integrates the Squeeze-and-Excitation (SE) attention mechanism [22] to enable dynamic modulation of semantic channel weights, thereby enhancing the network’s modeling capability for different defect targets through an interactive residual fusion mechanism. As shown in Figure 6, the IAFM adopts a dual-branch input architecture, where the inputs $X_{0}$ and $X_{1}$ originate from the outputs of different feature layers and thus encode complementary semantic information at different hierarchical levels. The module performs adaptive alignment to handle potential discrepancies in channel dimensionality. During the semantic aggregation stage, a joint feature representation is constructed via channel concatenation as $X_{\mathrm{cat}}=Concat(X_{0},X_{1})$, thereby forming a compact yet information-rich embedding that serves as the basis for subsequent adaptive weighting.

In the adaptive weighting stage, the SE module first compresses the spatial information of each channel in the joint feature representation via global average pooling to generate a global semantic descriptor, which is mathematically expressed as:

(13) $$z_{i}=\frac{1}{H\cdot W}\sum_{m=1}^{H}\sum_{n=1}^{W}X_{cat,i}(m,n)$$

where $Z_{i}$ denotes the global semantic value of the i-th channel, and H and W are the height and width of the input feature map, respectively. A bottleneck-structured fully connected layer is then applied to the compressed semantic descriptor to perform sequential dimensionality reduction and expansion to learn the importance weight of each semantic channel, which is expressed as:

(14) $$w=\sigma \left(W_{2}\cdot \delta \left(W_{1}z\right)\right)$$

where $W_{1}$ and $W_{2}$ are the weight matrices for the dimensionality reduction and expansion operations, respectively, δ(⋅) represents the ReLU activation function, and σ(⋅) denotes the Sigmoid activation function. To enable independent control over the two input branches, the generated weight vector w is partitioned according to the original channel counts, which is formulated as:

(15) $$w_{0},w_{1}=Split(w,[C_{0},C_{1}])$$

where the total length of w is $C_{0}+C_{1}$. The vector is divided into two parts such that $w_{0}$ contains the first $C_{0}$ weight values corresponding to the first branch, whereas $w_{1}$ contains the remaining $C_{1}$ weight values corresponding to the second branch, ensuring that each input branch is assigned an independent channel-wise weighting mechanism.

In the semantic reshaping stage, an interactive residual fusion mechanism is proposed. The mechanism first applies adaptive weighting independently to each of the two input branches, which is mathematically formulated as:

(16) $$\left\{\begin{array}{l} X_{0\_W}=X_{0}⊙w_{0}\\ X_{1\_W}=X_{1}⊙w_{1} \end{array}\right.$$

where ⊙ denotes element-wise multiplication, enabling precise per-channel weight modulation to accentuate salient features and suppress redundant information. To further facilitate inter-branch information exchange, cross-branch residual connections are then employed to realize deep feature interaction, where the original features of each branch are added to the weighted features of the other branch, as expressed:

(17) $$\left\{\begin{array}{l} Y_{0}=X_{0}+X_{1\_W}\\ Y_{1}=X_{1}+X_{0\_W} \end{array}\right.$$

This interactive design simultaneously preserves the original feature information of each branch and achieves complementary feature enhancement through cross-branch feature injection. As a result, heterogeneous semantic representations can mutually enhance each other, leading to more comprehensive and discriminative feature fusion. Finally, the two enhanced branches are concatenated to produce the fused output, which is formulated as:

(18) $$Output=Concat\left(Y_{0},Y_{1}\right)$$

where Concat denotes the channel-wise concatenation operation, yielding a fused feature representation that encapsulates the interactive information from both branches. Through adaptive semantic weighting and the proposed interactive residual fusion mechanism, the IAFM achieves effective integration of heterogeneous semantic information, reduces feature redundancy, suppresses complex background interference, and improves detection accuracy and robustness.

### 3.3. Progressive Shared-Weight Context Aggregation Module

The SPPF module in YOLOv11n performs spatial context aggregation through sequential multi-scale max-pooling operations. However, as an information compression mechanism, pooling inherently discards spatial detail during feature aggregation, which is particularly detrimental for defect detection tasks that require precise localization. Moreover, the pooling-based processing strategy of SPPF results in limited feature representation fidelity, while its fixed pooling operations lack the adaptability required for learning task-specific contextual information. To address these limitations, this paper proposes the Progressive Shared-Weight Context Aggregation (PSWCA) module as a replacement for the SPPF structure. The proposed module employs parameterized dilated convolutions in place of fixed pooling operations, enabling more expressive contextual modeling while preserving spatial details [23]. In addition, a progressive shared-weight mechanism is introduced to achieve parameter-efficient multi-scale context aggregation.

**Figure 7:**
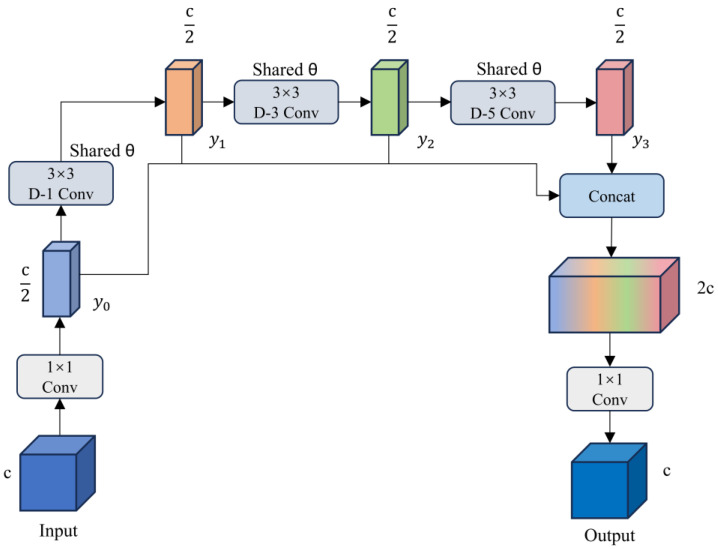
Architecture of the PSWCA module.

As shown in Figure 7, the PSWCA module adopts a progressive context aggregation architecture. The input feature x is first processed by a 1 × 1 convolution to reduce the channel dimensionality from C to C/2. A feature sequence $Y=[y_{0},y_{1},y_{2},y_{3}]$ is then constructed, where $y_{0}$ denotes the base feature after dimensionality reduction, and each subsequent feature $y_{i+1}$ is progressively generated from the preceding feature $y_{i}$ using a shared convolutional kernel applied at successively increasing dilation rates. Unlike the sequential pooling operations in SPPF, each processing step employs a learnable dilated convolution operating on the output of the previous step to perform context aggregation, thereby realizing progressive integration of contextual information from local to global scales.

The progressive shared-weight aggregation mechanism of the PSWCA module is formulated as:

(19) $$\left\{\begin{array}{l} y_{0}=Conv_{1\times 1}(x)\\ y_{1}=Conv_{3\times 3}(y_{0},\theta ,d=1)\\ y_{2}=Conv_{3\times 3}(y_{1},\theta ,d=3)\\ y_{3}=Conv_{3\times 3}(y_{2},\theta ,d=5)\\ Output=Conv_{1\times 1}\left(Concat\left[y_{0},y_{1},y_{2},y_{3}\right]\right) \end{array}\right.$$

where θ denotes the 3 × 3 convolutional kernel weights shared across all processing steps, d represents the dilation rate parameter, and $Conv_{3\times 3}(\cdot ,\theta ,d)$ denotes a dilated convolution operation parameterized by shared weights θ and dilation rate d. Each stage performs context refinement based on the output of the preceding step, enabling hierarchical context expansion from local structures to global semantics. Through the weight-sharing mechanism, a single convolutional kernel undertakes context aggregation over receptive fields of different spatial extents at varying dilation rates, thereby maintaining parameter efficiency.

Although SPPF and PSWCA both progressively enlarge the receptive field, their aggregation mechanisms differ. SPPF repeatedly applies a fixed max-pooling operator, which may retain similar dominant responses across pooling scales while discarding non-maximum spatial details. In contrast, PSWCA applies a shared learnable 3 × 3 kernel with increasing dilation rates, thereby varying the spatial sampling support without introducing separate scale-specific parameters. The resulting hierarchical features are concatenated and compressed through a 1 × 1 convolution, enabling learnable cross-scale mixing and down-weighting of overlapping responses. Therefore, PSWCA may mitigate, rather than completely eliminate, cross-scale feature redundancy while preserving complementary contextual information.

### 3.4. Wise-Inner-MPDIoU Loss Function

YOLOv11 adopts CIoU [24] as the bounding box regression loss function. Although CioU improves localization accuracy by jointly considering geometric factors such as overlap area, center point distance, and aspect ratio, it still faces three main challenges in steel surface defect detection: training instability caused by inconsistent annotation quality in defect datasets, limited adaptability of conventional IoU computation in complex detection scenarios, and insufficient geometric modeling of irregularly shaped defects under the existing geometric constraints. In industrial inspection datasets, defects with ambiguous boundaries or complex morphologies are often inconsistently annotated, and such low-quality samples introduce noisy gradients that compromise model training stability and hinder convergence.

Based on the above analysis, this paper first incorporates the Wise-IoU [25] mechanism from the perspective of data quality awareness to construct a quality-aware loss function. Wise-IoU dynamically evaluates and differentiates training samples according to their annotation quality. Through an adaptive weight adjustment mechanism, it reduces the influence of low-quality samples while reinforcing the learning signal from high-quality samples, thereby improving both training stability and bounding box regression accuracy.

Specifically, sample quality is quantified using an outlier degree indicator β, as:

(20) $$\beta =\frac{L_{IoU}^{*}}{L_{IoU}}\in [0,+\infty )$$

where $L_{IoU}^{*}$ is a constant derived from the variable $\bar{L_{IoU}}$, and $\bar{L_{IoU}}$ denotes the momentum running average of the historical loss values. Based on the outlier degree indicator β, Wise-IoU employs a dynamic non-monotonic focusing mechanism to adjust gradient allocation, as follows:

(21) $$r=\frac{\beta }{\delta \alpha^{\beta -\delta }}$$

where δ and α are hyperparameters, and r is the dynamic focusing coefficient. In all experiments, the dynamic focusing parameters were fixed at α=1.7 and δ=2.7. These two parameters were kept unchanged in the subsequent sensitivity experiments so that the influence of the auxiliary-box scale factor *ratio* could be examined independently. The final formulation of Wise-IoU is defined as:

(22) $$\left\{\begin{array}{l} L_{WIoU}=rR_{WIoU}L_{IoU}\\ R_{WIoU}=\exp\left(\frac{{\left(x-x_{gt}\right)}^{2}+{\left(y-y_{gt}\right)}^{2}}{{\left(W_{g}^{2}+H_{g}^{2}\right)}^{*}}\right)\\ L_{IoU}=1-IoU \end{array}\right.$$

where (x,y) and $\left(x_{gt},y_{gt}\right)$ denote the coordinates of the center points of the predicted box and the ground-truth box, respectively; $W_{g}$ and $H_{g}$ represent the width and height of the minimum enclosing box, respectively; and IoU denotes the Intersection over Union, measuring the overlap ratio between the predicted box and the ground-truth box. This mechanism assigns the highest gradient weight to samples with moderate outlier degrees while reducing the contribution of samples that are either too easy or too difficult, thereby mitigating the adverse effects of low-quality annotations and improving overall model performance.

Although Wise-IoU offers certain advantages in sample quality assessment, its primary focus lies in training process optimization, with relatively limited attention to the intrinsic computational properties of the IoU metric itself. To further improve the adaptability of conventional IoU in complex detection scenarios, Inner-IoU [26] optimizes the measurement process by introducing an auxiliary bounding box mechanism, which is formulated as:

(23) $$\left\{\begin{array}{l} b_{l}^{gt}=x_{c}^{gt}-\frac{w^{gt}\times ratio}{2},\;b_{r}^{gt}=x_{c}^{gt}+\frac{w^{gt}\times ratio}{2}\\ b_{t}^{gt}=y_{c}^{gt}-\frac{h^{gt}\times ratio}{2},\;b_{b}^{gt}=y_{c}^{gt}+\frac{h^{gt}\times ratio}{2}\\ b_{l}=x_{c}-\frac{w\times ratio}{2},\;b_{r}=x_{c}+\frac{w\times ratio}{2}\\ b_{t}=y_{c}-\frac{h\times ratio}{2},\;b_{b}=y_{c}+\frac{h\times ratio}{2} \end{array}\right.$$

where $\left(x_{c}^{gt},y_{c}^{gt}\right)$ and $\left(x_{c},y_{c}\right)$ denote the coordinates of the center points of the ground-truth box and the predicted box, respectively; $\left(w^{gt},h^{gt}\right)$ and (w,h) represent the width and height of the ground-truth box and the predicted box, respectively; and *ratio* is the scale factor ratio. Based on this formulation, the intersection and union areas of the two boxes are then computed as:

(24) $$IA=\left(\min(b_{r}^{gt},b_{r})-\max(b_{l}^{gt},b_{l})\right)\times \left(\min(b_{b}^{gt},b_{b})-\max(b_{t}^{gt},b_{t})\right)$$

(25) $$UA=(w^{gt}\times h^{gt})\times {\left(ratio\right)}^{2}+(w\times h)\times {\left(ratio\right)}^{2}-IA$$

The resulting Inner-IoU and its corresponding loss function are defined as:

(26) $$\left\{\begin{array}{l} IoU^{inner}=\frac{IA}{UA}\\ L_{Inner-IoU}=1-IoU^{inner} \end{array}\right.$$

The scale factor *ratio* controls the size of the auxiliary bounding boxes. When *ratio* = 1, the auxiliary boxes have the same scale as the original boxes; *ratio* < 1 produces smaller auxiliary boxes, whereas *ratio* > 1 produces larger ones. Since the appropriate auxiliary-box scale may depend on the overlap distribution of the training samples, *ratio* was treated as a tunable hyperparameter. Based on the sensitivity analysis presented in Section 4.4.4, *ratio* = 1.0 was adopted in the final model.

To address the limited adaptability of existing geometric constraints for irregularly shaped defects on steel surfaces, conventional area-overlap metrics exhibit insufficient capability in modeling complex defect morphologies. To improve geometric modeling for such scenarios, MPDIoU [27] introduces a corner-point distance constraint to strengthen shape-aware regression. Unlike conventional methods that rely solely on global overlap between bounding boxes, this formulation incorporates fine-grained geometric relationships at the corner level by penalizing the Euclidean distances between corresponding corner points of the predicted and ground-truth boxes:

(27) $$MPDIoU=IoU-\frac{d_{1}^{2}}{W_{\mathrm{img}}^{2}+H_{img}^{2}}-\frac{d_{2}^{2}}{W_{\mathrm{img}}^{2}+H_{img}^{2}}$$

where the distance terms are defined as:

(28) $$\left\{\begin{array}{l} d_{1}^{2}={\left(x_{1}^{prd}-x_{1}^{gt}\right)}^{2}+{\left(y_{1}^{prd}-y_{1}^{gt}\right)}^{2}\\ d_{2}^{2}={\left(x_{2}^{prd}-x_{2}^{gt}\right)}^{2}+{\left(y_{2}^{prd}-y_{2}^{gt}\right)}^{2} \end{array}\right.$$

where $W_{\mathrm{img}}$ and $H_{\mathrm{img}}$ denote the width and height of the input image, respectively, and are used to normalize the corner-point distance terms. $d_{1}^{2}$ and $d_{2}^{2}$ denote the squared Euclidean distances between the corresponding top-left and bottom-right corner points of the predicted and ground-truth boxes, respectively. This corner-point distance constraint facilitates improved geometric modeling of complex-shaped defects. Compared with conventional center-point constraints, corner-point constraints provide richer shape descriptors, thereby enhancing the adaptability of the geometric penalty to irregularly shaped defects.

The three aforementioned mechanisms are organically integrated by first constructing Inner-MPDIoU, which simultaneously improves the adaptability of IoU computation through auxiliary bounding boxes and strengthens geometric modeling for complex-shaped defects, as expressed:

(29) $$L_{\text{Inner-MPDIoU}}=L_{\mathrm{MPDIoU}}+IoU-IoU^{\mathrm{inner}}$$

The constructed Inner-MPDIoU is subsequently combined with the Wise-IoU quality-aware mechanism, replacing the original IoU computation component of Wise-IoU, to form the final composite loss function Wise-Inner-MPDIoU, as given:

(30) $$L_{\text{Wise-Inner-MPDIoU}}=rR_{\mathrm{WiseIoU}}L_{\text{Inner-MPDIoU}}$$

This fusion design realizes three-level collaborative optimization: the quality-aware mechanism directs training resources toward high-quality samples; the auxiliary bounding box mechanism improves the adaptability of IoU computation; and the corner-point distance constraints enhance geometric modeling for complex-shaped defects. The three mechanisms are mutually complementary and jointly contribute to improved detection accuracy and robustness.

## 4. Experimental Results and Analysis

### 4.1. Experimental Setup

All experiments were conducted on a Windows operating system equipped with an 11th Gen Intel^®^ Core™ i5-11400H processor at 2.70 GHz and an NVIDIA GeForce RTX 3050 GPU. The deep learning environment was built using PyTorch 2.2.2, with CUDA 12.1 and Python 3.10.14. The hyperparameter settings used for model training are summarized in Table 1.

### 4.2. Experimental Datasets

**Figure 8:**
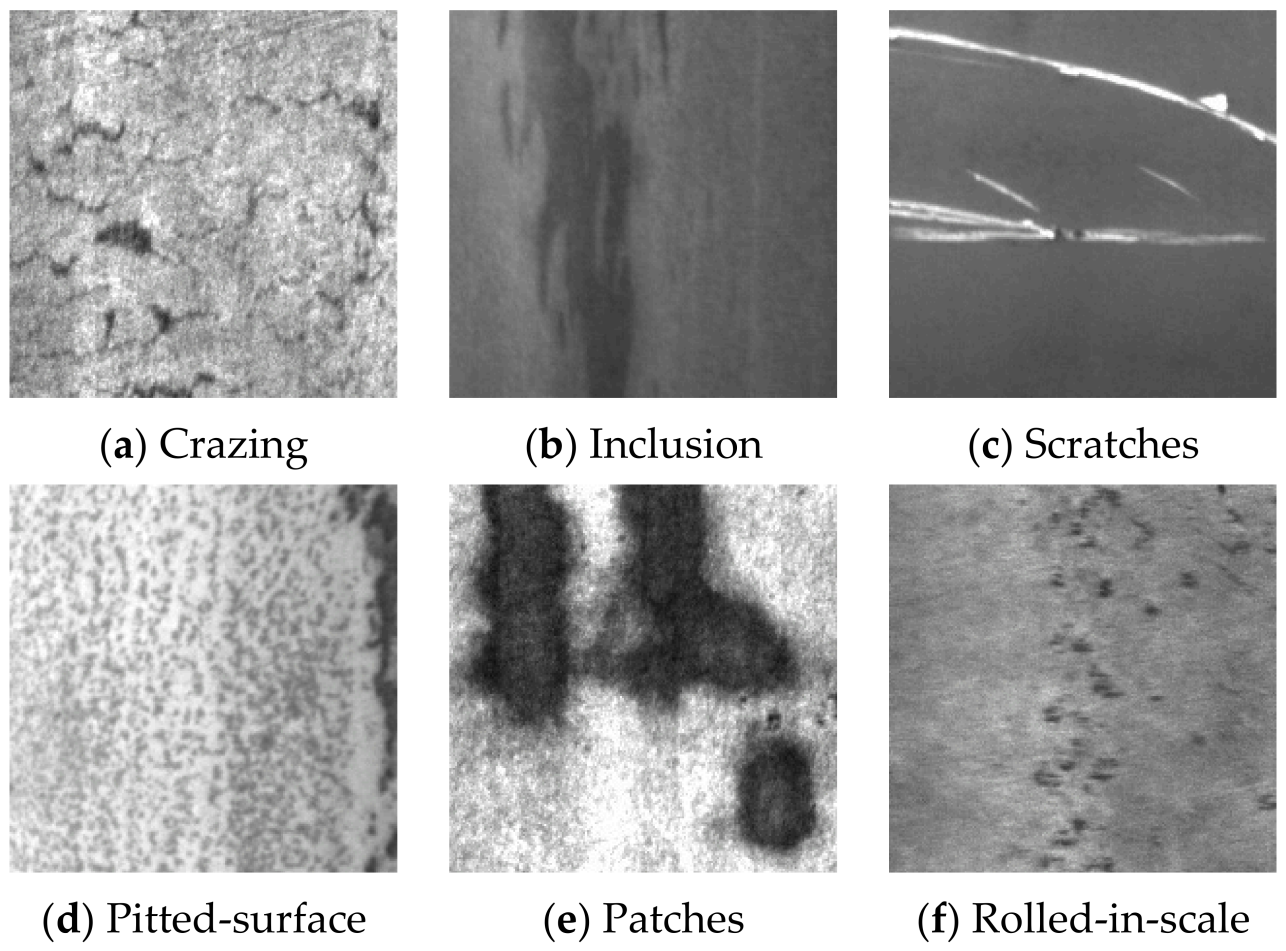
Representative images of different steel surface defect categories.

The experiments were conducted on the NEU-DET dataset [28], a widely used open-source benchmark for steel surface defect detection provided by Northeastern University. The dataset encompasses six categories of common steel surface defects: crazing, inclusion, scratches, pitted surface, patches, and rolled-in scale, which cover the principal defect categories encountered in industrial production. The dataset comprises a total of 1800 images, with 300 images for each defect category. Following common practice, the dataset was randomly partitioned into training, validation, and test sets in a ratio of 8:1:1. Representative samples of the defect categories are shown in Figure 8.

**Figure 9:**
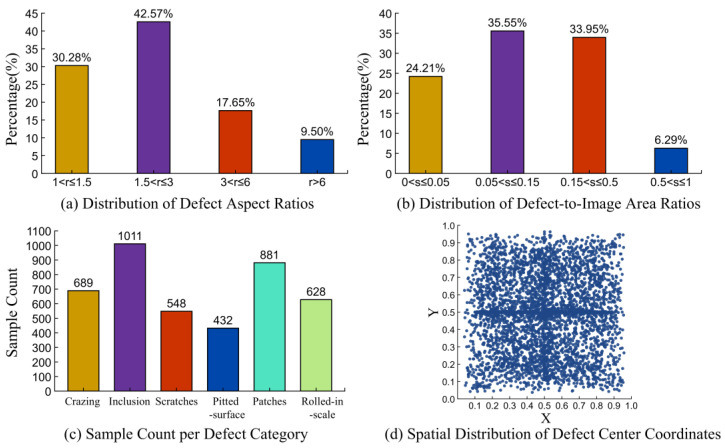
Statistical analysis of defect samples.

To further characterize the NEU-DET dataset, statistical analyses were conducted in terms of defect geometry, relative scale, category distribution, and spatial location, as shown in Figure 9. The representative examples in Figure 8 and the aspect-ratio distribution in Figure 9a indicate considerable variation in defect shape and elongation across different categories and instances. Figure 9b shows that small-area defects account for a large proportion of the annotations, while defects occupying larger image regions are also present, demonstrating a broad distribution of defect scales. Figure 9c reveals variation in the number of annotated instances among defect categories, whereas Figure 9d shows that defect centers are distributed across different regions of the image plane rather than concentrated in a fixed area. In this study, defect heterogeneity therefore refers to the inter-class and intra-class variability in morphology, relative scale, category frequency, and spatial location. Such variability increases the difficulty of learning a unified feature representation that remains discriminative across diverse defect types.

### 4.3. Evaluation Metrics

To assess the performance of the improved model, this study employs Precision (P), Recall (R), Average Precision (AP), mean Average Precision (mAP), the number of parameters (Params), floating-point operations (GFLOPs), and frames per second (FPS). Among these metrics, mAP50, defined as the mean AP across all defect categories at an IoU threshold of 0.5, is adopted as the primary indicator of overall detection accuracy. Params and GFLOPs are used to characterize model complexity, whereas FPS is used to evaluate practical inference efficiency. FPS was measured on an NVIDIA GeForce RTX 3050 GPU using an input resolution of 640 × 640 and a batch size of 1, with all model configurations evaluated under the same inference settings. The evaluation metrics are defined as follows:

(31) $$P=\frac{TP}{TP+FP}$$

(32) $$R=\frac{TP}{TP+FN}$$

(33) $$AP=\int_{0}^{1}P(r)dr$$

(34) $$mAP=\frac{1}{N}\sum_{i=1}^{N}{\mathrm{AP}}_{i}$$

(35) $$FPS=\frac{N_{\mathrm{img}}}{T_{\inf}}$$

where TP denotes the number of correctly detected defect instances, FP denotes the number of incorrect positive detections, and FN denotes the number of missed defect instances. AP represents the area under the precision–recall curve, whereas mAP is the arithmetic mean of the AP values across all N defect categories. $N_{\mathrm{img}}$ denotes the total number of images processed during runtime evaluation, and $T_{\inf}$ denotes the corresponding total inference time in seconds. A higher FPS value indicates greater inference throughput under the specified hardware and evaluation settings.

### 4.4. Proposed Module Experiments

#### 4.4.1. C3k2_MSD Module

To validate the effectiveness of the C3k2_MSD module and determine its optimal deployment configuration, comparative experiments were conducted on the NEU-DET dataset with the module placed at different network positions. Three configurations were evaluated: Model B, in which only the C3k2 modules in the backbone network were replaced; Model C, in which only the C3k2 modules in the neck network were replaced; and Model D, in which the C3k2 modules in both the backbone and neck networks were replaced simultaneously. The experimental results are reported in Table 2.

The results demonstrate that Model B achieves performance comparable to the baseline, indicating that introducing the module exclusively in the backbone network yields no significant performance gain. Model C improves mAP50 by 0.7 percentage points compared with the baseline, confirming the effectiveness of the module in the feature fusion stage of the neck network. Model D achieves the best overall performance, attaining an mAP50 of 77.1%, which represents a 1.0 percentage-point improvement over the baseline, while reducing the parameter count to 2.4 M. This indicates a favorable trade-off between detection accuracy and computational efficiency. These findings suggest that the C3k2_MSD module plays a pivotal role in the feature fusion process of the neck network, and that deploying it in both the backbone and neck networks ensures consistency of the multi-scale mechanism, enabling cross-level feature enhancement.

The training-resource results indicate that C3k2_MSD introduces additional overhead during training. Compared with the baseline, Models B, C, and D increase the average training time per epoch by 16.9%, 20.2%, and 38.5%, respectively, while peak GPU memory changes from 1.41 GB to 1.54 GB, 1.42 GB, and 1.56 GB. Model D incurs the largest time overhead because C3k2_MSD is deployed in both the backbone and neck. This additional cost mainly arises from retaining the parallel Conv–BN branches and their intermediate activations within DRB_5_ and DRB_7_ for gradient computation. The non-monotonic memory variation may reflect differences in feature-map dimensions and activation lifetimes across deployment locations. During deployment, the internal branches of DRB_5_ and DRB_7_ are reparameterized into equivalent 5 × 5 and 7 × 7 depthwise convolutions, reducing their branch-level inference overhead. Overall, the results indicate a trade-off between increased training-resource consumption and richer multi-scale representation, with only a modest increase in peak GPU memory under the evaluated settings.

#### 4.4.2. IAFM

To validate the superiority of the IAFM, comparative experiments were conducted on the NEU-DET dataset based on the YOLOv11n baseline model. The proposed module was benchmarked against several mainstream feature fusion networks, including BiFPN [29], GFPN [30], AFPN [31], HSFPN [32], and RFPN [33]. The comparative results are presented in Table 3.

As shown in Table 3, the IAFM achieves the highest detection accuracy among all compared methods, attaining an mAP50 of 77.2%, representing a 1.1 percentage-point improvement over the baseline. Compared with GFPN, IAFM achieves superior detection accuracy while reducing the parameter count by 25% and the computational cost by approximately 10%, demonstrating more favorable overall performance. Although HSFPN and BiFPN are more lightweight in terms of parameter count, their mAP50 values of 76.5% and 76.8%, respectively, remain lower than that of IAFM. These results demonstrate that the IAFM module achieves the best trade-off among detection accuracy, parameter efficiency, and computational complexity, which can be attributed to its three-stage fusion architecture and interactive residual mechanism.

To further investigate the internal design of IAFM, additional comparative experiments were conducted to evaluate the effectiveness of different attention mechanisms used in the adaptive weighting stage. Specifically, the SE attention mechanism was compared with SimAM [34], ELA [35], CA [36], MLCA [37], and EMA [38]. The experimental results are presented in Table 4.

As shown in Table 4, the SE attention mechanism achieves the highest detection accuracy among all compared methods, attaining an mAP50 of 77.2%. In terms of model complexity, SE maintains a parameter count of 2.7 M and a computational cost of 7.4 GFLOPs, both of which remain within a reasonable range compared with the other methods. Through channel-wise weight learning, SE can effectively identify the critical channels carrying defect-related information, demonstrating superior adaptability under the complex background conditions of steel surface images. Considering both detection accuracy and computational efficiency, the SE attention mechanism achieves a favorable performance balance, validating its suitability for the adaptive weighting stage of the IAFM.

#### 4.4.3. PSWCA Module

To validate the effectiveness of the dilation rate combination [1, 3, 5] employed in the PSWCA module, comparative experiments were conducted to evaluate the impact of different dilation rate configurations on context aggregation performance. The progressive shared-weight architecture was held constant across all configurations, and multiple representative dilation rate combinations were selected for comparative analysis. The experimental results are reported in Table 5.

As shown in Table 5, the dilation rate combination [1, 3, 5] achieves the highest detection accuracy, attaining an mAP50 of 77.6%. Except for the PS and RS defect categories, this configuration also achieves the highest AP values across all remaining categories. Compared with the [1, 3, 7] configuration, mAP50 improves by 0.2 percentage points, indicating that a third dilation rate of 5 is more suitable for context aggregation. Among the remaining configurations, [1, 3, 9], [1, 2, 4], and [1, 2, 6] all yield lower mAP50 values than [1, 3, 5]. In particular, compared with the [1, 5, 7] configuration, mAP50 improves by 2.9 percentage points, further demonstrating the effectiveness of the progressive context aggregation configuration of [1, 3, 5]. These results confirm the rationality and effectiveness of the selected dilation rate combination.

It should be noted that weight sharing directly reduces parameter duplication, whereas the progressively varied dilation rates and the final 1 × 1 fusion may mitigate overlapping feature responses across scales. Since Table 5 evaluates detection performance rather than inter-level feature correlation, the proposed design is described as reducing, rather than eliminating, feature redundancy.

#### 4.4.4. Loss Function Comparison and Scale-Factor Sensitivity Analysis

To validate the effectiveness of the Wise-Inner-MPDIoU loss function, comparative experiments were conducted on the NEU-DET dataset, benchmarking it against several mainstream loss functions, including CIoU, PIoU [39], SIoU [40], and EIoU [41]. The objective was to evaluate the effect of different loss functions on model detection performance. The experimental results are reported in Table 6.

As shown in Table 6, the proposed Wise-Inner-MPDIoU loss function achieves the best overall performance among all compared methods, yielding the highest precision (0.789), recall (0.754), and mAP50 (80.2%). Compared with conventional IoU-based loss functions, the proposed loss demonstrates clear advantages in both localization accuracy and detection robustness. The improved performance can be attributed to the complementary integration of the quality-aware weighting mechanism of Wise-IoU, the auxiliary bounding box mechanism of Inner-IoU, and the corner-point distance constraint of MPDIoU, which jointly improve bounding box regression quality. These results confirm the effectiveness of the proposed loss function in improving detection performance for steel surface defects.

To evaluate the sensitivity of Wise-Inner-MPDIoU to the auxiliary-box scale, *ratio* was varied from 0.5 to 1.5 with an interval of 0.1. The dynamic focusing parameters were fixed at α=1.7 and δ=2.7, while the network architecture and all other training settings remained unchanged. The corresponding results are presented in Table 7.

As shown in Table 7, *ratio* = 1.0 achieves the highest mAP50 and mAP50:95 values of 0.802 and 0.475, respectively, while providing relatively balanced precision and recall values of 0.789 and 0.754. Although *ratio* = 0.9 and *ratio* = 1.2 obtain comparable mAP50:95 values of 0.470 and 0.474, respectively, their overall detection results are lower than those obtained with *ratio* = 1.0. The performance variation is not strictly monotonic, but larger deviations from 1.0 generally lead to lower overall detection performance. These results suggest that Wise-Inner-MPDIoU exhibits moderate sensitivity to the auxiliary-box scale within the evaluated range, and *ratio* = 1.0 provides the most favorable overall balance under the present experimental settings.

### 4.5. Ablation Study

To validate the effectiveness of each proposed module, ablation experiments were conducted on the NEU-DET dataset using YOLOv11n as the baseline. The experimental results are presented in Table 8.

The experimental results validate that each proposed module contributes positively to detection performance. When C3k2_MSD is introduced individually, mAP50 improves by 1.0 percentage point to 77.1%, while the parameter count is reduced to 2.4 M. The IAFM improves mAP50 by 1.1 percentage points, although this comes with an increase in computational cost to 7.4 GFLOPs. The PSWCA module achieves a 1.5 percentage-point performance gain with no increase in computational cost, highlighting the advantage of the progressive shared-weight mechanism. When C3k2_MSD and IAFM are combined, mAP50 reaches 78.1%, a further improvement over either module used in isolation, demonstrating the strong compatibility between multi-scale feature extraction and adaptive feature fusion. When all three modules are combined, mAP50 further increases to 79.4%, representing a 3.3 percentage-point improvement over the baseline, while the parameter count and computational cost increase modestly to 2.7 M and 6.6 GFLOPs, respectively.

Upon incorporating the Wise-Inner-MPDIoU loss function into the three-module network, mAP50 increases from 79.4% to 80.2%, while precision and recall improve from 0.768 and 0.710 to 0.789 and 0.754, respectively. Because Wise-Inner-MPDIoU is applied only during training and does not modify the inference architecture, the three-module configuration and the complete model achieve the same inference speed of 133.14 FPS. Compared with the baseline speed of 146.27 FPS, the complete CCP-YOLO model maintains 133.14 FPS while improving mAP50 by 4.1 percentage points. Overall, these results suggest that the proposed architecture and loss-function modifications provide a favorable balance among detection accuracy, model complexity, and practical inference efficiency under the evaluated setting.

It should be noted that improvements in the aggregate mAP50 do not necessarily imply uniform gains across all defect categories, because different modules may affect defect representations differently depending on their morphology, scale, and visual characteristics.

**Figure 10:**
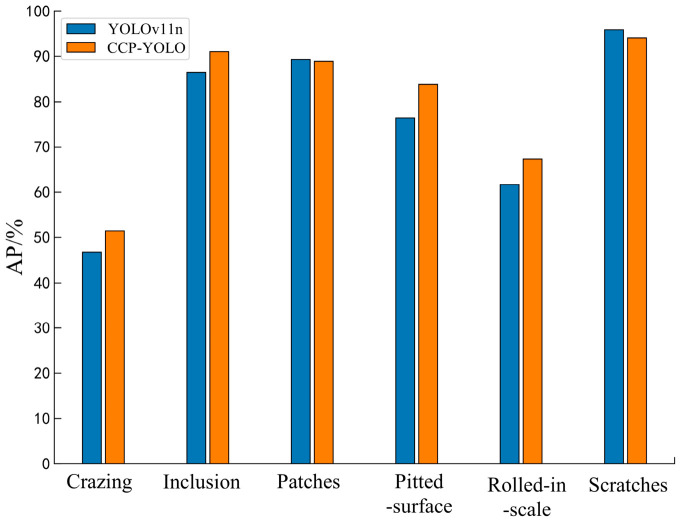
Comparison of per-category average precision (AP) between the proposed algorithm and the baseline model.

Figure 10 presents the per-category AP comparison between CCP-YOLO and the baseline YOLOv11n on the NEU-DET dataset. The largest improvement is observed for rolled-in scale, with an AP gain of 9.1 percentage points. Crazing, pitted surface, inclusion, and patches improve by 5.6, 5.1, 3.6, and 1.5 percentage points, respectively. In contrast, the AP of scratches decreases marginally by 0.1 percentage points. This small category-specific variation may be related to the elongated and narrow appearance of scratches. Although the proposed multi-scale and contextual modules enhance broader feature representation, the aggregation of larger receptive-field information may slightly dilute fine linear responses or increase interference from visually similar background textures. Because the decrease is limited and the current ablation results report aggregate rather than class-wise module effects, it cannot be reliably attributed to a single component. Therefore, this result is interpreted as a minor category-specific trade-off rather than a systematic degradation of detection performance.

### 4.6. Comparative Experiment Analysis

To evaluate the detection performance of the proposed model, comparative experiments were conducted by benchmarking CCP-YOLO against mainstream object detection algorithms across five evaluation metrics: precision, recall, detection accuracy, model parameter count, and computational complexity. The experimental results are reported in Table 9.

The results demonstrate that CCP-YOLO achieves superior overall performance across multiple evaluation metrics. In terms of precision and recall, the proposed model attains 0.789 and 0.754, representing improvements of 2.1 and 8.2 percentage points over the YOLOv11n baseline, respectively. Moreover, the mAP50 reaches 80.2%, corresponding to a 4.1 percentage-point improvement over the baseline, while maintaining a reasonable parameter count and computational complexity. Compared with other lightweight models, the mAP50 of CCP-YOLO surpasses those of YOLOv8n, YOLOv10n, and YOLOv12n by 4.3, 4.7, and 4.2 percentage points, respectively.

Further comparative analysis indicates that some models, such as YOLOv5s, achieve detection accuracy comparable to the baseline level but require substantially higher parameter counts and computational costs, making them less suitable for lightweight deployment. In contrast, ultra-lightweight models such as YOLOv9-t offer reduced parameter counts but exhibit considerably lower detection accuracy. Although RT-DETR-r18 employs an advanced Transformer-based architecture, its 19.8 M parameters and 57.0 GFLOPs impose a computational burden far beyond that of lightweight detectors, while its detection accuracy remains substantially below that of CCP-YOLO. Benefiting from the proposed multi-level collaborative optimization strategy, CCP-YOLO achieves significant accuracy improvements upon a lightweight architectural foundation, thereby striking a favorable balance between detection performance and computational efficiency.

### 4.7. Visualization Comparison

**Figure 11:**
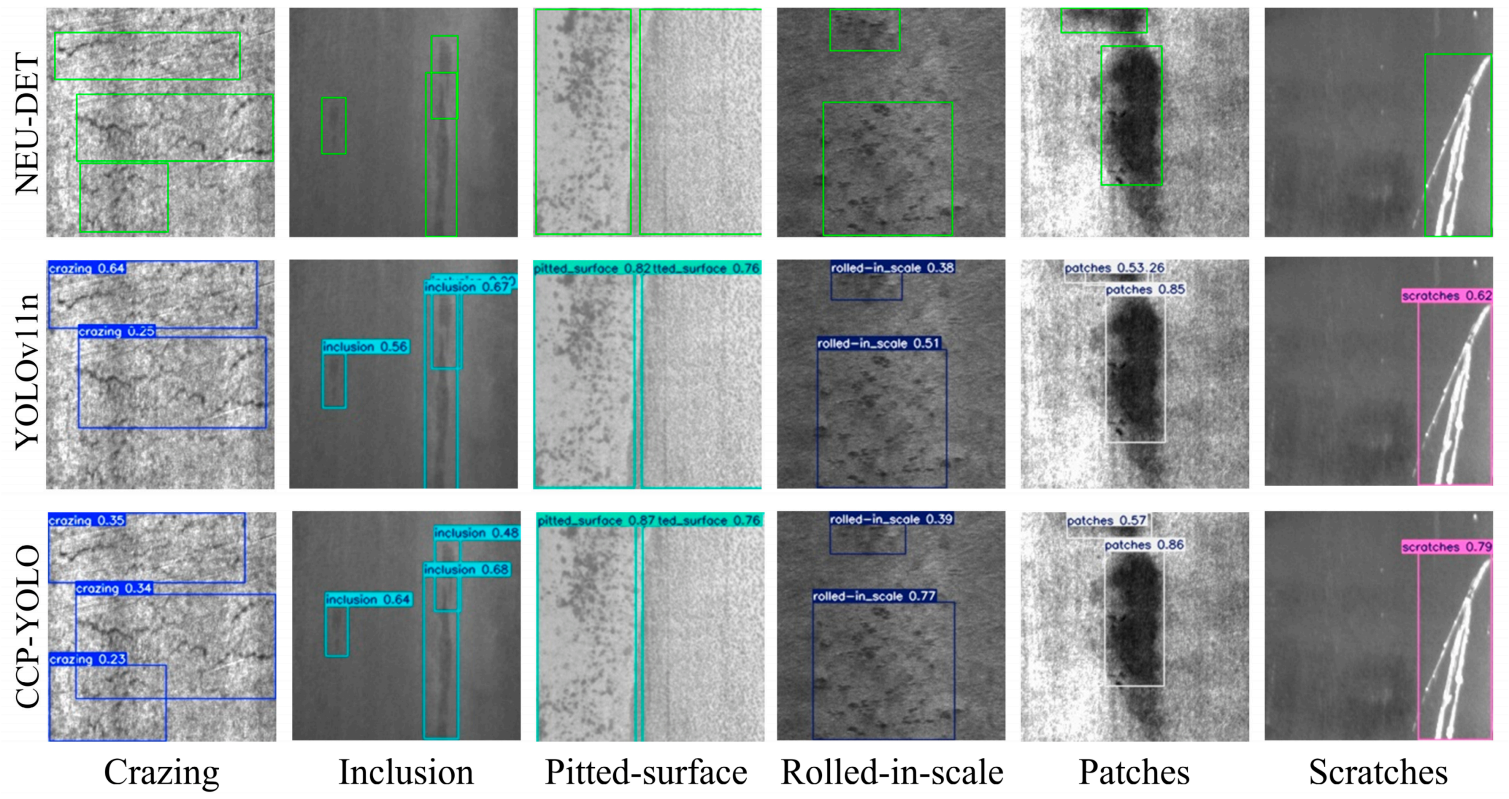
Visual comparison of detection results between CCP-YOLO and YOLOv11n.

To qualitatively evaluate the detection performance of CCP-YOLO, representative samples from the NEU-DET test set were selected for visual comparison against the baseline YOLOv11n, as shown in Figure 11. The baseline model exhibits localization deviations and false positive detections in complex defect scenarios, including spurious detections in background regions for scratches and missed detections for crazing and pitted surface defects. In contrast, CCP-YOLO demonstrates consistently superior detection performance on the same test samples by effectively reducing false positives in background regions and missed detections while improving localization accuracy. The visualization results further confirm the synergistic effect of the proposed network architecture improvements and the loss function modification.

**Figure 12:**
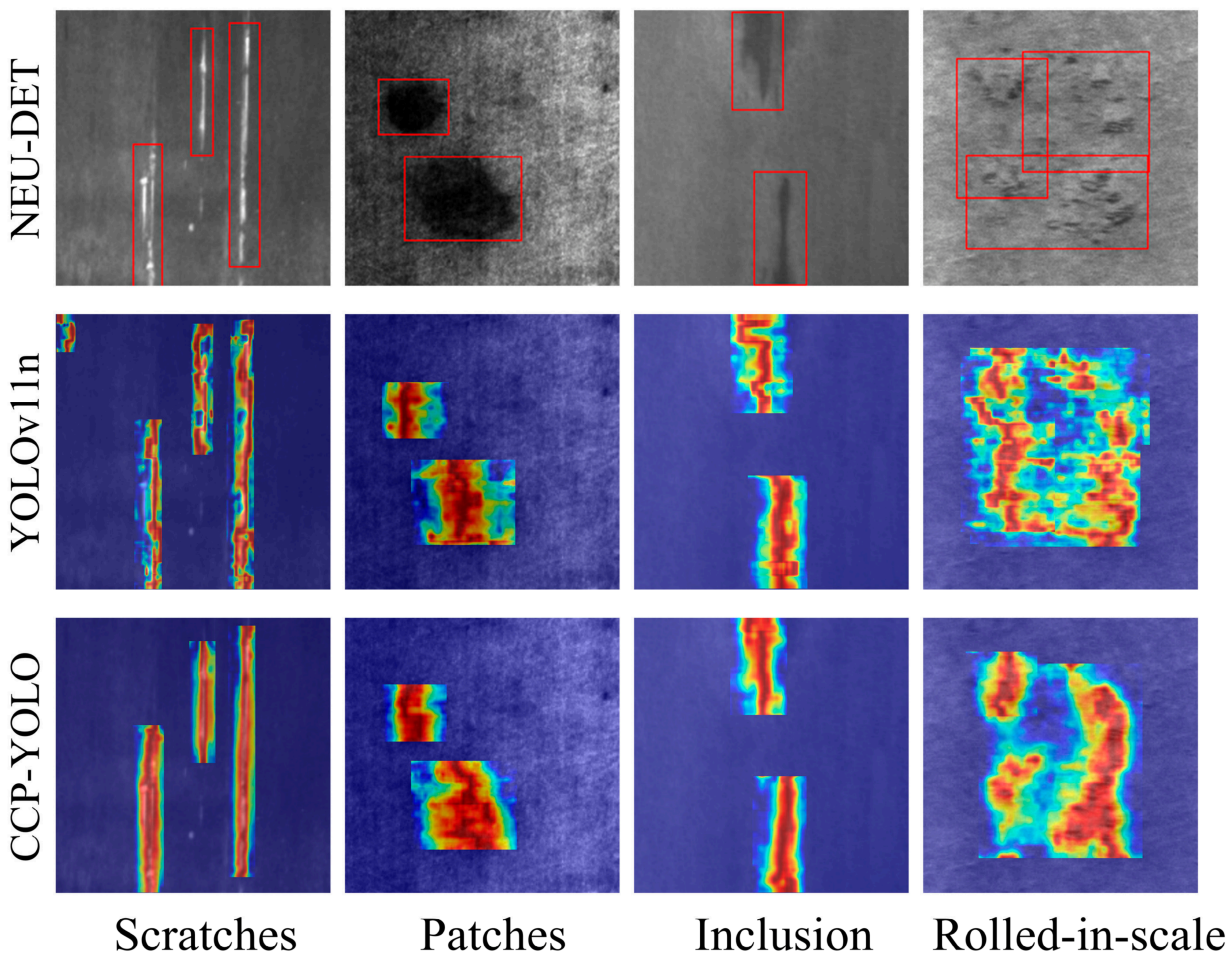
Heatmap visualization comparison between CCP-YOLO and YOLOv11n.

To further analyze the feature attention mechanism of CCP-YOLO, gradient-weighted class activation mapping (Grad-CAM) visualization was employed to visualize the critical response regions during the model’s detection process, with the corresponding results shown in Figure 12. Compared with the baseline model, CCP-YOLO exhibits more concentrated and precise activation regions for the majority of defect targets, with a more rational weight distribution that enables effective focus on the discriminative feature regions of defects. By contrast, the baseline YOLOv11n suffers from dispersed attention regions or attention shift on certain defect categories. The Grad-CAM visualization results demonstrate the improvements achieved by CCP-YOLO in defect feature extraction and target localization.

### 4.8. Generalization Validation

To evaluate the generalization capability of CCP-YOLO, validation experiments were conducted on the GC10-DET dataset [42], another widely used benchmark for steel surface defect detection. The GC10-DET dataset comprises 2294 grayscale images spanning 10 surface defect categories, including punching hole, foreign object, weld line, silk spot, water spot, indentation, crease, oil spot, waist folding, and crescent gap. For consistency with the previous experiments, the dataset was randomly partitioned into training, validation, and test sets in a ratio of 8:1:1. Owing to the substantial differences between GC10-DET and NEU-DET in terms of defect categories and image characteristics, GC10-DET provides an effective benchmark for evaluating the cross-dataset generalization capability of the proposed method. The experimental results are reported in Table 10.

CCP-YOLO achieves an mAP50 of 71.5% on the GC10-DET dataset, representing a 3.7-percentage-point improvement over the YOLOv11n baseline. Recall increases from 0.638 to 0.711, whereas precision decreases slightly from 0.691 to 0.679. This asymmetric change suggests that the proposed model detects a larger proportion of defect instances under the shifted data distribution, but may also produce a modest increase in false-positive predictions. The imbalance may be related to differences between NEU-DET and GC10-DET in defect categories, morphology, and image characteristics. Overall, these results provide evidence of the cross-dataset generalization potential of CCP-YOLO, while indicating that false-positive suppression under domain shift remains an area for further improvement.

## 5. Conclusions

In this work, a novel steel surface defect detection model named CCP-YOLO is proposed based on the YOLOv11n framework to address key challenges in industrial defect detection, including difficulties in multi-scale feature extraction, limited effectiveness of heterogeneous feature fusion, loss of spatial detail information, and insufficient adaptability of bounding box regression loss functions. The proposed method integrates four targeted improvements: (1) the C3k2_MSD Multi-Scale Dilated Reparameterization feature extraction module, designed to enhance multi-scale representation capability; (2) the IAFM Interactive Adaptive Feature Fusion Module, which improves cross-layer feature interaction and fusion efficiency; (3) the PSWCA Progressive Shared-Weight Context Aggregation module, aimed at strengthening contextual information modeling while preserving parameter efficiency; and (4) the Wise-Inner-MPDIoU loss function, which integrates Wise-IoU, Inner-IoU, and MPDIoU to improve bounding box regression quality. Through these components, the proposed method achieves collaborative optimization of both the network architecture and the loss function.

Experiments conducted on the NEU-DET dataset demonstrate that CCP-YOLO attains an mAP50 of 80.2%, representing a 4.1 percentage-point improvement over the baseline YOLOv11n model. The proposed method also improves precision and recall while maintaining a favorable balance between detection accuracy, computational cost, and inference efficiency, achieving 133.14 FPS under the evaluated hardware setting. Ablation studies support the contribution of the proposed modules, while the per-category analysis indicates adaptability to defects with different morphological and scale characteristics. In addition, comparative experiments against mainstream detection algorithms demonstrate competitive overall performance, and validation on the GC10-DET dataset provides further evidence of cross-dataset generalization potential.

Despite the observed improvements, CCP-YOLO still has certain architectural limitations when handling severely occluded or densely overlapping defect instances. The proposed C3k2_MSD, IAFM, and PSWCA modules mainly enhance multi-scale feature representation, cross-level feature fusion, and contextual aggregation, but they do not explicitly model occlusion relationships or instance-level separation. When adjacent defects exhibit similar textures or partially overlapping boundaries, their feature responses may interfere with each other, and highly overlapping predictions may be difficult to distinguish during localization and post-processing. In addition, although the Wise-Inner-MPDIoU loss improves bounding box regression, it may not fully compensate for the limited visual evidence available under severe occlusion. Introducing dedicated occlusion-aware interaction or boundary-sensitive instance discrimination mechanisms may improve performance in such cases, but could also increase computational and memory costs, thereby affecting real-time deployment.

The experimental results indicate a potentially favorable balance between detection performance and computational complexity; however, the deployment efficiency of CCP-YOLO on resource-constrained edge devices has not yet been systematically evaluated. Future work will examine a staged model-compression and deployment strategy. First, structured channel pruning will be considered for convolutional layers in the backbone and neck, with channel importance estimated using weight- or activation-based criteria. Layer-wise sensitivity analysis will be conducted to determine suitable pruning ratios, followed by fine-tuning to mitigate potential accuracy degradation. Second, INT8 post-training quantization will be evaluated using a representative calibration subset. If the resulting accuracy degradation is non-negligible, quantization-aware training will be further investigated to improve the balance between numerical precision and deployment efficiency. Finally, the compressed model will be evaluated on representative edge platforms in terms of detection accuracy, inference latency, model size, peak memory consumption, and, where measurable, power consumption. These investigations will provide a more systematic basis for balancing detection performance and resource efficiency in practical steel surface inspection.

## Figures and Tables

**Table 1:** Experimental hyperparameter settings.

Hyperparameter	Value
Initial Learning Rate	0.01
Momentum	0.937
Weight Decay	0.0005
Batch size	8
Optimizer	SGD
Image size	640 × 640
Epochs	400

**Table 2:** Performance, complexity, and training-resource comparison of C3k2_MSD at different network locations.

Configuration	mAP50/%	Params/M	GFLOPs	Average Training Time/Epoch (s)	Peak GPU Memory (GB)
Baseline	76.1	2.5	6.3	24.09	1.41
B	76.1	2.5	6.3	28.15	1.54
C	76.8	2.5	6.4	28.95	1.42
D	77.1	2.4	6.4	33.36	1.56

**Table 3:** Performance comparison of different feature fusion networks.

Method	mAP50/%	Params/M	GFLOPs
Baseline	76.1	2.5	6.3
BiFPN	76.8	1.9	6.3
GFPN	77.1	3.6	8.2
AFPN	76.0	2.6	8.8
HSFPN	76.5	1.8	5.6
RFPN	76.1	2.5	6.3
IAFM	77.2	2.7	7.4

**Table 4:** Performance comparison of different attention mechanisms in IAFM.

Attention Mechanism	mAP50/%	Params/M	GFLOPs
SimAM	76.6	2.6	6.5
ELA	74.5	5.8	6.9
CA	76.4	2.7	6.5
MLCA	76.9	2.6	6.5
EMA	76.7	2.7	8.2
SE	77.2	2.7	7.4

**Table 5:** Performance comparison of the PSWCA module with different dilation rate configurations.

Config	AP/%						mAP50/%
	Cr	In	Pa	PS	RS	Sc	
[1, 3, 7]	44.2	81.6	91.3	87.2	68.9	91.1	77.4
[1, 3, 9]	42.9	90.0	90.7	83.6	60.0	94.0	76.9
[1, 5, 7]	37.8	91.1	89.6	82.7	54.9	91.6	74.7
[1, 2, 4]	45.2	87.1	91.0	74.9	60.1	94.6	75.5
[1, 2, 6]	44.3	89.6	88.1	75.7	67.1	93.2	76.3
[1, 3, 5]	45.6	91.1	92.0	81.8	60.0	94.8	77.6

**Table 6:** Comparison results of loss functions.

Loss Function	P	R	mAP50/%
CIoU	0.768	0.710	79.4
PIoU	0.743	0.744	78.9
SIoU	0.751	0.748	79.4
EIoU	0.716	0.728	77.5
Wise-CIoU	0.721	0.744	78.3
Wise-DIoU	0.760	0.705	78.1
Wise-Inner-MPDIoU	0.789	0.754	80.2

**Table 7:** Sensitivity analysis of the auxiliary-box scale factor *ratio* in Wise-Inner-MPDIoU.

*Ratio*	P	R	mAP50/%	mAP50:95/%
0.5	0.745	0.721	78.0	46.7
0.6	0.728	0.706	76.3	42.0
0.7	0.787	0.724	77.5	45.1
0.8	0.737	0.728	77.4	46.3
0.9	0.729	0.743	78.7	47.0
1.0	0.789	0.754	80.2	47.5
1.1	0.758	0.714	77.9	46.4
1.2	0.706	0.722	77.6	47.4
1.3	0.725	0.730	76.7	46.0
1.4	0.758	0.717	77.7	46.3
1.5	0.775	0.686	77.0	45.1

**Table 8:** Ablation study results of the proposed modules.

C3k2_MSD	IAFM	PSWCA	Wise-Inner-MPDIoU	P	R	mAP50/%	Params/M	GFLOPs	FPS
				0.768	0.672	76.1	2.5	6.3	146.27
√				0.716	0.723	77.1	2.4	6.4	129.52
	√			0.737	0.729	77.2	2.7	7.4	141.68
		√		0.716	0.747	77.6	2.7	6.3	138.32
			√	0.751	0.701	76.0	2.5	6.3	146.27
√	√			0.726	0.743	78.1	2.6	6.4	128.19
√	√	√		0.768	0.710	79.4	2.7	6.6	133.14
√	√	√	√	0.789	0.754	80.2	2.7	6.6	133.14

**Table 9:** Performance comparison of different detection algorithms.

Method	P	R	mAP50/%	Params/M	GFLOPs
YOLOv3-tiny	0.672	0.659	69.4	9.5	14.3
YOLOv5n	0.700	0.735	75.3	2.1	5.8
YOLOv5s	0.719	0.717	76.2	7.8	18.7
YOLOv6n	0.700	0.731	75.1	4.1	11.5
YOLOv7-tiny	0.671	0.685	72.4	6.0	13.1
RT-DETR-r18	0.782	0.682	74.4	19.8	57.0
YOLOv8n	0.715	0.733	75.9	2.6	6.8
YOLOv8s	0.745	0.727	75.3	9.8	23.4
YOLOv9-t	0.655	0.696	73.6	1.7	6.4
YOLOv10n	0.772	0.652	75.5	2.6	8.2
YOLOv11n	0.768	0.672	76.1	2.5	6.3
YOLOv12n	0.699	0.696	76.0	2.6	6.3
Proposed Method	0.789	0.754	80.2	2.7	6.6

**Table 10:** Generalization validation results on the GC10-DET dataset.

Models	P	R	mAP50/%	Params/M	GFLOPs
YOLOv11n	0.691	0.638	67.8	2.5	6.3
Proposed Method	0.679	0.711	71.5	2.8	6.6

## Data Availability

The datasets used in this study are publicly available. The NEU-DET and GC10-DET datasets can be accessed through their respective official websites. Additional experimental data generated during this study are available from the corresponding author upon reasonable request.
